# A solution and practice for combining multi-source heterogeneous data to construct enterprise knowledge graph

**DOI:** 10.3389/fdata.2023.1278153

**Published:** 2023-09-28

**Authors:** Chenwei Yan, Xinyue Fang, Xiaotong Huang, Chenyi Guo, Ji Wu

**Affiliations:** ^1^School of Computer Science (National Pilot Software Engineering School), Beijing University of Posts and Telecommunications, Beijing, China; ^2^Key Laboratory of Trustworthy Distributed Computing and Service (BUPT), Ministry of Education, Beijing University of Posts and Telecommunications, Beijing, China; ^3^School of Economics and Management, Tsinghua University, Beijing, China; ^4^Department of Electronic Engineering, Tsinghua University, Beijing, China

**Keywords:** knowledge graph construction, heterogeneous data, knowledge graph update, enterprise knowledge graph, graph database

## Abstract

The knowledge graph is one of the essential infrastructures of artificial intelligence. It is a challenge for knowledge engineering to construct a high-quality domain knowledge graph for multi-source heterogeneous data. We propose a complete process framework for constructing a knowledge graph that combines structured data and unstructured data, which includes data processing, information extraction, knowledge fusion, data storage, and update strategies, aiming to improve the quality of the knowledge graph and extend its life cycle. Specifically, we take the construction process of an enterprise knowledge graph as an example and integrate enterprise register information, litigation-related information, and enterprise announcement information to enrich the enterprise knowledge graph. For the unstructured text, we improve existing model to extract triples and the F1-score of our model reached 72.77%. The number of nodes and edges in our constructed enterprise knowledge graph reaches 1,430,000 and 3,170,000, respectively. Furthermore, for each type of multi-source heterogeneous data, we apply corresponding methods and strategies for information extraction and data storage and carry out a detailed comparative analysis of graph databases. From the perspective of practical use, the informative enterprise knowledge graph and its timely update can serve many actual business needs. Our proposed enterprise knowledge graph has been deployed in HuaRong RongTong (Beijing) Technology Co., Ltd. and is used by the staff as a powerful tool for corporate due diligence. The key features are reported and analyzed in the case study. Overall, this paper provides an easy-to-follow solution and practice for domain knowledge graph construction, as well as demonstrating its application in corporate due diligence.

## 1. Introduction

A comprehensive understanding of enterprises and their associations is vital in the financial domain, as it benefits several real-world applications, such as fraud detection, risk management, corporate due diligence. To this end, the knowledge graph is often employed as a useful tool to provide information. In particular, enterprise knowledge graphs have attracted wide attention and application from academia and industry as an important infrastructure of artificial intelligence. Among financial firms, it has become an increasing trend to use knowledge graphs to represent knowledge and information. The Caixin company proposed the Caituan Knowledge Graph, ^1^ which mainly depicts the investment and holding relationships between enterprises. The Tonghuashun^2^ company constructed an enterprise knowledge graph to show the upstream and downstream supply and demand relationships of enterprises in the industry supply chain. The China Merchants Bank introduced a knowledge graph of enterprise association relationships to display a variety of inter-enterprise relationships (Jia, 2022). Numerous studies and applications have shown that knowledge graphs can form an effective structured representation of knowledge, have rich semantic information representation capabilities and flexible graph structures, and are effective carriers of knowledge and information.

Although a lot of research has been conducted on knowledge graphs in academia and industry (Liu et al., 2023a,b), there are still many challenges associated with domain knowledge graph construction and application. The first challenge is the richness of the domain knowledge graph and the multi-source nature of the data. Different sources of data provide different perspectives on entities and their relationships. However, this also increases the difficulty, as it is different to process multi-source heterogeneous data, especially for unstructured data. The second challenge, a common issue in practical knowledge deployment and application, is determining how to store and retrieve the data efficiently. These two problems are commonly faced in the process of domain knowledge graph construction and subsequent deployment and application.

Thus, we propose a complete process framework for constructing an enterprise knowledge graph that combines structured data and unstructured data, which includes data processing, information extraction, knowledge fusion, data storage, and update strategies aiming at improving the quality of the knowledge graph, extend its life cycle, and providing a solution for enterprise knowledge graph construction, deployment, and application. The main contributions are as follows.

- We propose a solution for the construction of enterprise knowledge graphs, taking the enterprise knowledge graph as an example. The whole process, including deployment and application, provides decision-making support for both academic research and industry application.
- We focus on the extraction of unstructured text when combining multi-source heterogeneous data into a knowledge graph and employ and improve the CasRel model (Wei et al., 2020) to extract entities and relations. The source code has been publicly available on Github at https://github.com/chenwei23333/EKG. Additionally, the text processing approaches and storage strategies are well-designed according to each type of data.
- To ensure that the knowledge graph is up-to-date, we summarize two strategies for updating the information, i.e., passive updates and active updates. The latter refers to updates manually triggered by humans to fulfill the partial data updates.
- We study a Corporate Due Diligence System based on a constructed enterprise knowledge graph and summarize its features as a case study, which can provide single-point queries, group enterprises queries, etc. to support decision-making. This system has been put into use in HuaRong RongTong (Beijing) Technology Co., Ltd. to assist the business staff in generating corporate due diligence reports.

## 2. Related works

### 2.1. Enterprise knowledge graph

Knowledge graphs can be divided into two categories: General Knowledge Graphs (GKGs) and Domain Knowledge Graphs (DKGs). Well-known GKGs, such as Freebase (Bollacker et al., 2007) and DBpedia (Bizer et al., 2009), focus on knowledge coverage, which means that the number of entities and relations is massive. The applications that GKGs could support are also varied, including search engines, intelligent question-answering, personalized recommendations, etc. (Qi et al., 2017). Compared with GKGs, DKGs contain smaller numbers of entities and relations. Meanwhile, their application scenarios are abundant and need to be customized to meet different business needs (Xu et al., 2016).

DKGs are widely used in medical (Li et al., 2020; Gong et al., 2021), financial (Song et al., 2017; Zhan and Yin, 2018; Chen and Xiang, 2020; Mao et al., 2022), scholarly research (Liu J. et al., 2020; Zhou et al., 2020; Kanakaris et al., 2021), tourism (Gao et al., 2020), disaster prevention (Du et al., 2020), e-government (Promikyridis and Tambouris, 2020), and other fields to provide a structured network knowledge base for the corresponding professionals. The data source of DKGs generally comes from richly preserved professional data in the domain, including structured data, semi-structured data, and unstructured data, such as free text and images. For example, medical knowledge graphs are usually derived from electronic medical records (Gong et al., 2021), drug information (Wishart et al., 2017), or other medical data as auxiliary systems to reduce the diagnosis burden of doctors and allow better clinical decisions to be made. Science and technology knowledge graphs (Zhou et al., 2020) mainly utilize multi-source data related to the field, such as scientific papers, patents, and scientific projects, to help researchers find partners and grasp trends in academic research.

In the financial field, the most commonly used knowledge graph (KG) is the enterprise knowledge graph. An enterprise KG refers to a vertical domain knowledge graph that focuses on corporate information and relationships, and it has wide commercial applications. One of the major applications of the enterprise KG is the general collection of enterprise information, especially enterprise risk information. Chen and Xiang (2020) constructed an enterprise KG that uses several public internet financial information websites as data sources to monitor the operating situation and risk information of enterprise entities in the market. Song et al. (2017) implemented an enterprise search engine using corporate tax information, corporate risk rating information, legal document information, news, and other information. In addition to the general enterprise graph, more applications focus on segmented business. The nodes and relationships that these graphs focus on, while still being enterprise-centric, need to be integrated into specific application scenarios. For example, some studies use the enterprise KG for stock price predictions. In addition to enterprise entities, they also introduce “concepts” (such as the blockchain) and “industries” (such as manufacturing) as nodes (Long et al., 2020) that are more in line with the information that the stock market is interested in Jin et al. (2019) constructed a loan knowledge graph of small and micro enterprises, added attributes, such as the “stakeholder” and “contact information” to the enterprise KG nodes, and computed the fraud probability of each enterprise.

It can be seen that, although the existing EKGs select the enterprise as the main focus, the definitions of nodes, relationships, and node attributes are very flexible, which requires the joint participation of technical personnel and business personnel as well as customization according to different business scenarios. At the same time, determining how to ensure true integration with a business is also a key consideration throughout the whole process of building the DKG. Most of the above-mentioned applications of the enterprise KG are still in the primary stage, and there is a relatively large gap between KG technology and actual business logic, which limits the capabilities of the enterprise KG.

### 2.2. Knowledge graph construction pattern

The construction pattern of the knowledge graph mainly includes the top-down pattern and the bottom-up pattern. Top-down refers to first defining the ontology and data schema of the knowledge graph according to the characteristics of the knowledge and then organizing entities and relations based on the pre-defined schema. As the domain knowledge graph is mainly used to assist complex business analyses and support decision-making, the typical domain-specific scenarios and the background of the personnel must be considered during knowledge graph design. This puts forward clear requirements for the construction and application of knowledge graphs. As a result, the current mainstream construction of domain knowledge graphs is top-down.

Chen and Xiang (2020) utilized a top-down mode to construct an enterprise risk knowledge graph for an intelligent question-answering chatbot. When building the ontology layer, the object attributes (subsidiaries, holdings, etc.) and data attributes (company name, staff number, etc.) owned by the company ontology are defined. When building the data layer, the semi-structured enterprise information data are obtained by parsing the source code of the company's home page. Open-source tools are used for named entity recognition and dependency parsing, and an algorithm based on a twin neural network is used for entity alignment. Lv et al. (2020) used equity data to analyze shareholding relationships and ratios among financial institutions and constructed a knowledge graph of financial equities.

Different from the top-down mode, the requirement of the bottom-up pattern is to first extract entities and relations from the data, organize and summarize the entities to form bottom-level concepts, gradually abstract upwards to form the upper-level concepts, and finally generate the ontology schema and the KG. Song et al. (2017) used a bottom-up approach to construct an enterprise knowledge graph for question-answering systems. The source data were taken from enterprise databases, public tax data, public risk rating data, legal data, and news. Hybrid algorithms based on rules and machine learning are used to extract both entities and relations from structured, semi-structured, and unstructured data, and the SVM is used for entity linking. However, the schema-less construction method leads to looseness in the data structure and increases the consumption of unified management. This makes it necessary to introduce Elasticsearch to support full-text fuzzy retrieval in addition to standard RDF triples storage.

Referring to the construction process of the existing domain knowledge graph, we selected the top-down construction method in order to maximize the use of expert knowledge, improve the efficiency of data storage, and facilitate later updates and maintenance.

### 2.3. Knowledge graph update

The total amount of knowledge will continue to increase over time, so the construction process of the knowledge graph is supposed to be constantly and iteratively updated (Brenas and Shaban-Nejad, 2021). Otherwise, the immutability of knowledge graphs will shorten their life cycles rapidly. The updating of the knowledge graph includes not only the updating of the concept/ontology layer but also the updating of the data layer (Liu et al., 2016; Ma et al., 2019).

The updating of the data layer is the key concern in enterprise knowledge graphs, since the relationships between enterprises are always changing, such as when a partnership breakdown or legal person alteration happens (Tang et al., 2019). While the entities remain unchanged, the triples in the KG are updated with news snippets; that is, the necessary link-adding or link-removing operations are performed to ensure that the KG is up-to-date. Fang et al. (2020) added time-series-related features to the knowledge graph construction process to let users know the last modified time. However, this lacks an update method guided by users, i.e., user-centered timely update.

In the investigation of the updating of domain knowledge graphs in industrial applications, it was found that most knowledge graphs adopt a regular update schema, and users cannot actively apply for updates. To tackle this problem, this paper proposes two update strategies, active updates and passive updates, to improve the dynamics and real-time nature of the knowledge graph. Specifically, the strategy is based on regular updates, supplemented by active updates from users, so that users can clearly understand whether the queried data are up-to-date.

## 3. Overview of knowledge graph construction

Knowledge graph construction (KGC) is a complicated process, especially in the case of multi-source heterogeneous data, which mainly includes preparing data, designing the data schema, extracting entities and relations, storing data, updating data, and implementing specific applications according to business logic. Thus, our goal is to propose an approach to construct an enterprise knowledge graph by integrating structured, semi-structured, and unstructured data, which is easy to follow and adaptable to similar research.

**Figure 1:**
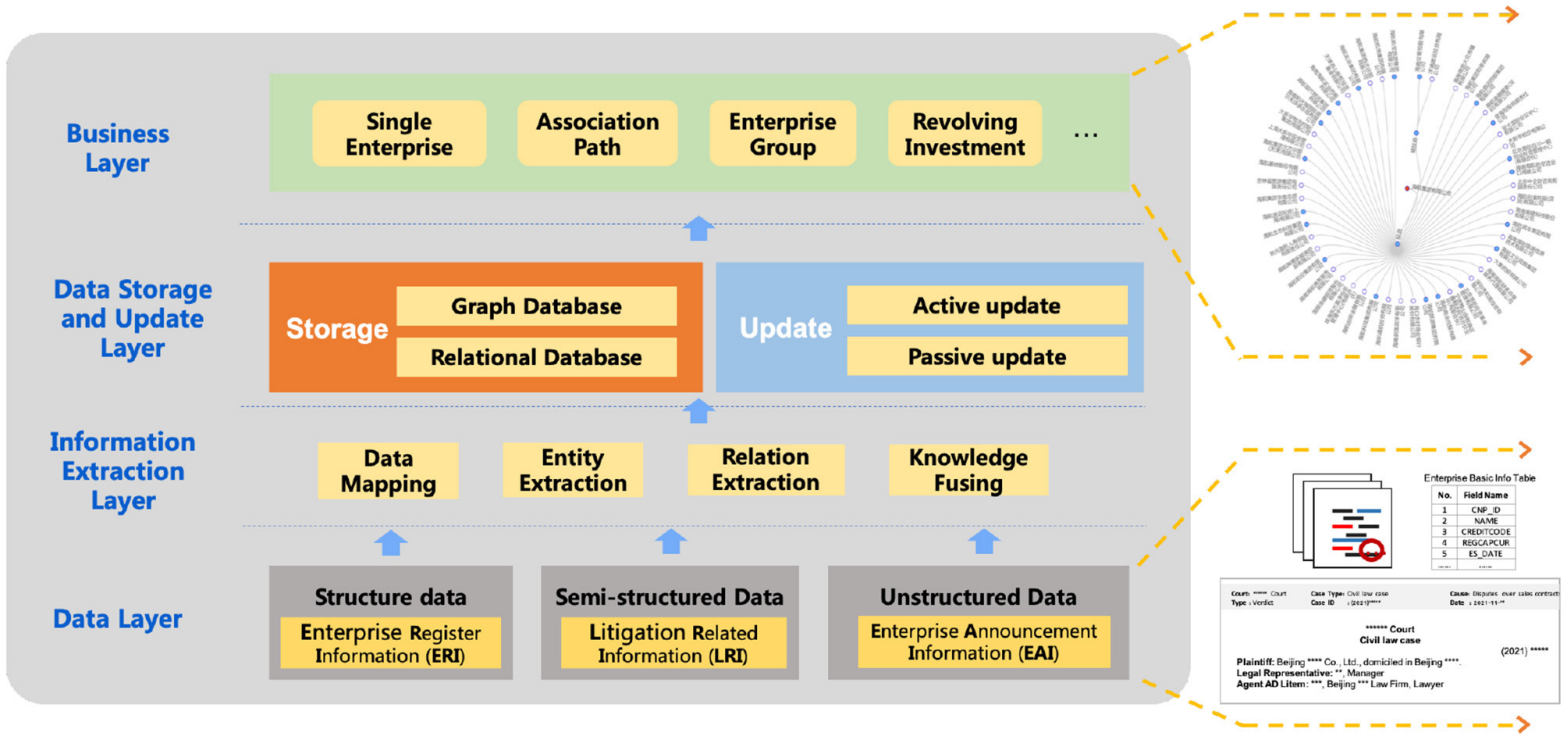
The whole enterprise knowledge graph construction framework. The bottom right part displays three sources of data, while the upper right part displays a retrieval result for one enterprise and its associated entities.

We summarize the key points in the construction process in Figure 1 and list five key research questions Q1–Q5 as follows.

**Q1**: Is it necessary to utilize multi-source data when constructing enterprise KG?
**Q2**: How to extract entities and their relations from unstructured text effectively?
**Q3**: How to solve the data conflicts from different data source?
**Q4**: How to select proper database to store data?
**Q5**: When and how to update the data in the KG?

Next, based on the whole enterprise KG construction framework in Figure 1, we introduce the details of each layer in the following sections and answer each question.

## 4. KGC: data preparation

### 4.1. Multi-source heterogeneous data

For **Q1**: Is it necessary to utilize multi-source data when constructing enterprise KG, the answer is definitely yes. Multi-source data are essential for constructing an enterprise knowledge graph, since they can provide information from different aspects. The key points are to identify what kind of information is needed and to further define where to collect it. Generally speaking, commonly-used data sources include basic information about the enterprise, announcements, annual reports, news, court judgments, and administrative penalties related to the enterprise, etc. The selection of data sources is strongly related to the application scenario of the enterprise knowledge graph. Due to our downstream task being limited to corporate due diligence, which will be introduced in detail in Section 9.1, we chose three types of data, enterprise register information (ERI), litigation-related information (LRI), and enterprise announcement information (EAI), to provide background information and find potential enterprise risks. The detailed information is shown in Table 1.

**Table 1:** Detailed information about the three types of data used to construct our enterprise knowledge graph.

**Type**	**Data**	**Description**
Structured	Enterprise register information (ERI)	Refers to data registered in the Industrial and Commercial Bureau, such as the scope of business, registered capital, etc.
Semi-structured	Litigation-related information (LRI)	Refers to records of litigation relationships between an enterprise and other individuals or enter-prises during its business activities.
Unstructured	Enterprise announcement information (EAI)	Refers to public announcements, which are collected from the Shanghai Stock Exchange Announcement of Oriental Fortune.

According to our research, these data can paint a basic picture of an enterprise and its potential risks. ERI data pay more attention to the basic information about the enterprise itself, such as the enterprise name, enterprise unified credit code, enterprise branch information, etc., and describe the attributes of the enterprise from multiple dimensions. LRI data and EAI data emphasize relationships between the enterprise and other enterprises/persons, such as investment and litigation relationships. Specifically, EAI data contain rich information, which may reveal the operating profits, changes in senior executives, equity pledges, and cooperation projects, where we can dig out the enterprise situations and the relationships between enterprises.

### 4.2. Schema design

Schema design is the basis of constructing a knowledge graph that identifies ontologies, and relationships. Generally, compared with the general knowledge graph (GKG), the entity classes and relationships in the domain KG are more clear. For example, in our proposed enterprise KG, the key entity classes are person and enterprise, and the numbers of entities and relationships in the enterprise KG are also less than in the open GKG. Therefore, we adopt a top-down construction pattern (Yang et al., 2018); that is, we first identify the KG schema and then organize the data matching the schema into the KG.

**Figure 2:**
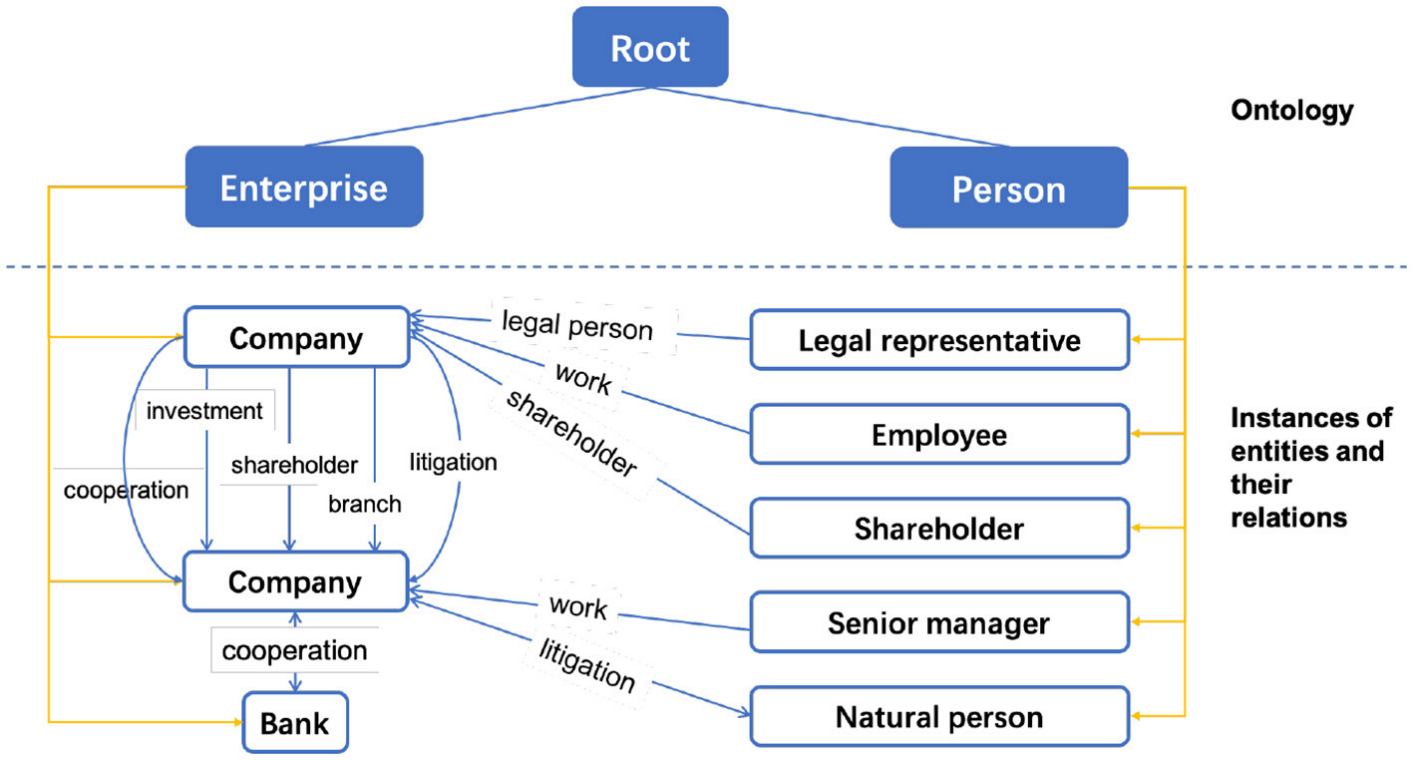
The schema design for our enterprise knowledge graph. The key entity classes are person and enterprise. We further subdivided these two entity classes into finer-grained types (for the convenience of displaying relationships, we listed two company instances).

In all, after the data analysis and expert evaluation, our enterprise KG defines two kinds of entities and seven relationships, as shown in Figure 2. The first kind of entity is the enterprise entity, and the other is the person entity. It should be noted that although we only present two kinds of entities in the schema, the entity can be divided into a finer-grained type, e.g., company, bank. To distinguish them, we add an attribute for the entity type to record the fine-grained label. Similarly, the person entity sets legal representatives, shareholders, senior managers, employees, and natural persons as its attribute values. The relations are investment, branch, shareholder, litigation, cooperation, work, and legal person.

## 5. KGC: information extraction

Information extraction is mainly applied to semi-structured and unstructured data to obtain the entities and their relations from text. Next, we introduce the methods used for each type of data in detail.

### 5.1. Information extraction for semi-structured data

Semi-structured data are a widely used data source for constructing a KG, and they require a pre-defined frame design and information extraction. The advantage is that the structure of these data is more fixed than free text, and the extraction process can be realized with the help of tags in the data. For the enterprise KG, the process of extracting litigation relations from verdict documents can be summarized as follows:

1. Data pre-processing and field selection. The first step is to clean the text and segment the body of the verdicts document. This is implemented by cutting sentences based on HTML tags and removing empty sentences. For the enterprise KG, the body field is selected to extract the litigation pairs.
2. Extract entities. The second step is to extract the relevant entities of the verdict document from the “parties” field.
3. Extract relations. The third step is to extract litigation relationship data. The pre-defined litigation pair keywords are plaintiff–defendant, appellant–appellee, and execution application–person subject to execution. However, there are dozens of keywords in the actual data. Therefore, if no sentence is retrieved by the keywords, the verdict document will be recorded in the log so that it can be manually checked to add the missing keywords.
4. Construct sets. After the relevant sentence of the litigation relationship has been extracted, the relevant entities extracted from the party's field are used to match the sentence to construct the source entities set and the target entities set.

The extraction process can be summarized by the following steps. First, according to the observation and analysis of a large amount of data, the keywords of the litigation relationship are all located at the beginning of the segmented sentence, so the first keyword-matching method is used for sentence screening, and the sentences related to the litigation relationship are selected. Then, we collect the source entities and target entities from these sentences. Finally, the triples are generated.

### 5.2. Information extraction for unstructured data

Now, we move to **Q2**: How to extract entities and their relations from unstructured text effectively? It is more complicated to extract entities and relations from unstructured data. Due to the error accumulation problem in pipeline methods, we prefer to use joint extraction methods to extract entities and relations. Furthermore, we consider the overlapping problem in the announcement text, i.e., multiple relationships simultaneously exist in a pair of entities or multiple triples share the same entity. Thus, we exploit a joint extraction method for entities and relations, named CasRel (Wei et al., 2020), and improve it based on the domain needs. Next, we introduce the model structure and validate it on our dataset.

#### 5.2.1. Model

**Figure 3:**
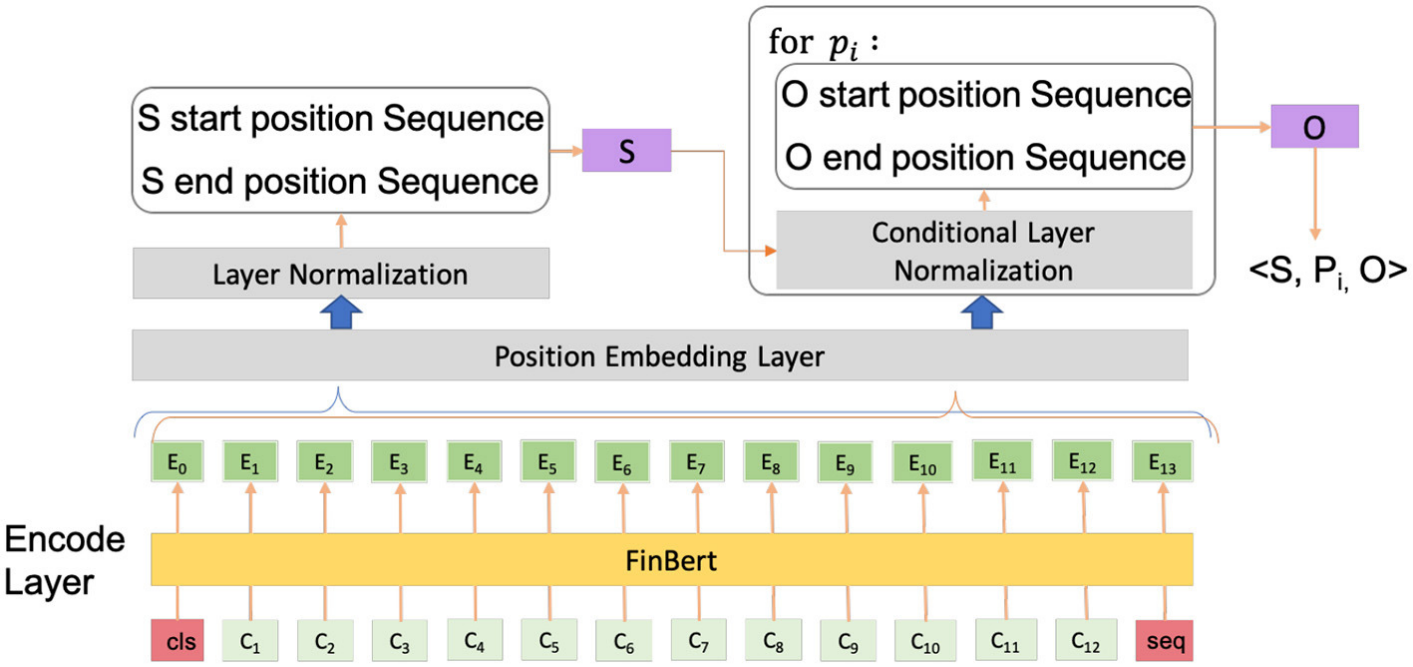
The whole structure of our model. For each sentence, the model generates triples like (subject, predicate, object).

CasRel models the relationships as mappings from head to tail entities and generates triples like (Subject, Predicate, Object). To obtain a more credible result, we improve it from two aspects: (1) We replace the pre-trained language model with FinBERT (Liu Z. et al., 2020); and (2) We add a position layer to add position embeddings to each token embedding. Compared to the previous works, such as Li et al. (2021), which focused on the decoding reformation of CasRel, our improvement comes from the encoding perspective. The whole model structure is shown in Figure 3. In short, we extract all subjects (*S*) form the text and extract each corresponding object (*O*) for the case of every relation (*P*). The specific pre-defined entities and relationships can be seen in the data preparation section.

For the first layer, the encode layer, the pre-trained language model FinBERT is utilized to convert the original text sequence *S* = {*x*_1_, *x*_2_, ..., *x*_*n*_} of the announcement into word indices. Then, we obtain the vector representation *w*_*j*_ of the word *x*_*j*_ in the sentence. Formally, the output of the first layer is denoted as


(1) $$\begin{array}{l} h_{j}^{0}=x_{j} \end{array}$$

Although BERT (Devlin et al., 2019) achieved SOTA results on many NLP tasks, FinBERT is more targeted at the financial field. It uses the same structure as BERT in terms of the network structure, but the pre-trained corpus for FinBERT includes financial news from the last ten years, various public research reports and company announcements, and Chinese financial encyclopedia text. In addition, during the task-level pre-training process of FinBERT, two supervised learning tasks are introduced, research report industry classification and financial entity recognition, so that the model can better learn knowledge about the financial field at the semantic level and also learn the features of words and sentences in the finance domain.

The second layer is the position embedding layer, which embeds the positional information of entities. The position embeddings are concatenated with the sentence representation as an extra feature in the next layer. Formally, the output of the second layer is denoted as


(2) $$\begin{array}{l} h_{j}^{1}=h_{j}^{0}+x_{j}^{p}, \end{array}$$

where $x_{j}^{p}$ is the positional embedding of the *j*-th token, where *p* represents the position index in the input sequence.

Next is layer normalization. The features are normalized according to the number of features in the text sequence, that is, the mean and variance of all features are rescaled. Here, we use two binary classifiers to predict the first and last positions of the subject. Formally, the potential subjects are predicted as


(3) $$\begin{array}{l} p_{j}^{start_{s}}=\sigma \left(W_{start}h_{j}^{N}+b_{start}\right), \end{array}$$

(4) $$\begin{array}{l} p_{j}^{end_{s}}=\sigma \left(W_{end}h_{j}^{N}+b_{end}\right), \end{array}$$

where $p_{j}^{start_{s}}$ and $p_{j}^{end_{s}}$ represent the probability of identifying the *j*-th word in the input sequence *S* as the start and end positions of a subject, respectively. $h_{j}^{N}$ is the result of layer normalization, *W*_*start*_ and *W*_*end*_ are the trainable weights, *b*_*start*_ and *b*_*end*_ are the bias, and σ is the sigmoid activation function.

The next step is conditional layer normalization. We randomly sample a labeled subject *S*, extract the embeddings of the beginning and end of subject *S* from the sequence, and concatenate them as the feature of subject *S*. During predictions, the traverse of all subjects can be used to replace random sampling. Then, we take the feature of subject *S* as a condition and make a conditional layer norm on the text sequence.

Finally, the sequence of the last layer is used to predict the corresponding *P* and *O* of subject *S*. For each relationship, we construct such a module to predict the first and last positions of the corresponding object, so that both the object and predicate are predicted at the same time. Formally, the potential objects are predicted as


(5) $$\begin{array}{l} p_{j}^{start_{o}}=\sigma \left(W_{start}^{r}\left(h_{j}^{CN}+v_{k}\right)+b_{start}\right), \end{array}$$

(6) $$\begin{array}{l} p_{j}^{end_{o}}=\sigma \left(W_{end}^{r}\left(h_{j}^{CN}+v_{k}\right)+b_{end}\right), \end{array}$$

where $p_{j}^{start_{s}}$ and $p_{j}^{end_{s}}$ represent the probability of identifying the *j*-th word in the input sequence *S* as the start and end positions of an object, respectively. $h_{j}^{CN}$ is the result of conditional layer normalization, and *v*_*k*_ is the vector representation of the *k*-th subject.

Formally, given sentence *x*_*j*_ from training set *D*, we aim to maximize the likelihood of training set *D*:


(7) $$\begin{array}{l} \overset{\left|D\right|}{\underset{j=1}{\prod }}p=\underset{s\in T_{j}}{\prod }p\left(s|x_{j}\right)\underset{r\in T_{j}}{\prod }p_{r}\left(o|s,x_{j}\right), \end{array}$$

where *p*(*s*|*x*_*j*_) represents the probability that *S* exists in the sentence when predicting the head entity *S* in the *j*-th sentence, and *p*_*r*_(*o*|*s, x*_*j*_) represents the probability of the existence of a tail entity *O* that has a relation *r* with the current header entity *S*.

#### 5.2.2. Experiment and results

To validate the model, we annotated 1,200 announcements manually. For each announcement, we annotated the existing entities and labeled the relations between them. Based on current needs and the existing dataset, we defined and labeled six relations including an equity pledge, equity transfer, investment, equity increase, equity reduction, and cooperation. Considering the small size of the dataset, the training set, test set, and validation set were randomly divided in the ratio of 4:1:1.

As for the parameters, the batch size was eight, and the maximum length of the input sequence was 256, which was defined by the statistics on the dataset. The classifier used the sigmoid activation function. Our model adopts an ADAM optimizer with a learning rate of 0.00001. Table 2 reports the results of the test set. CasRel (Wei et al., 2020), PRGC (Zheng et al., 2021), and TPLinker (Wang et al., 2020) were selected as our baseline models. In the meantime, we also trained a pipeline model on the current dataset, which consisted of a BiLSTM network for named entity recognition and a BiLSTM network for relation extraction. The F1 score of this pipeline model was 62.48%, which is considerably lower than that of other joint models. In addition, for simplicity, the ablation experiment is also listed in Table 2.

**Table 2:** The experimental results of the triple extraction and the ablation experiments to validate the FinBERT and position embedding (PE).

**Model**	**Precision (%)**	**Recall (%)**	**F1-score (%)**	**Recall on overlapped triples(%)**
BiLSTM + BiLSTM	69.33	56.85	62.48	–
TPLinker (Wang et al., 2020)	72.50	64.18	71.18	–
PRGC (Zheng et al., 2021)	74.83	54.87	63.31	26.47
CasRel (Wei et al., 2020)	73.64	66.47	69.55	41.14
CasRel (FinBERT)	78.44	65.17	71.20	42.86
CASREL (PE)	72.82	70.65	71.72	45.71
CasRel (FinBERT + PE)	76.80	69.15	72.77	42.86

Moreover, we evaluated the performance of the listed models on overlapping triples. Our statistical results indicate that there were 172 pieces of data in the dataset with overlapping triples. Here, we provide an instance of an overlapping triple in Table 3. As reported in the last column of Table 2, our model achieved similar results to CasRel and was significantly superior to PRGC.

**Table 3:** An instance of overlapping triples from the dataset.

**Sample**	**Ground truth**	**Wrong extraction**	**Omission**	**Over prediction**
In early August of this year, Company A announced the acquisition of 10% of the equity of Company C held by Company B for a price of CNY 29.7 million. At the end of August, Company D and Company E acquired 90% and 1% equity of Company F for a price of CNY 142 million, respectively. Both transactions have been completed.	(B, ET, A) (C, EF, A) (E, EF, A)	(B, ET, C) (C, EF, A) (E, EF, A)	(B, ET, A) (C, EF, A)	(B, ET, A) (C, EF, A) (E, EF, A) (D, EF, E)

“ET” in the triples refers to “equity transfer”.

Cooperation is a two-way relationship that often has clear keywords such as “strategic cooperation” and “signing an agreement” in the text, and the order of entities has little impact on semantics. Therefore, it is less difficult to extract the cooperative relationship from several relationships in the dataset, and there is no conflict between the cooperative relationship and other relationships in the graph database. Therefore, in the subsequent data fusion stage of our work, only the “cooperation” relationship was selected for merging into the enterprise knowledge graph. We also measured the effect of the entity relationship extraction of different models on the cooperative relationship in the test set, as shown in Table 4. The F1 score of the improved CASREL model was 84.62%.

**Table 4:** The experimental results of the relationship extraction of cooperation between enterprises.

**Model**	**Precision**	**Recall**	**F1-score**
CasRel	68.42%	92.85%	78.89%
CasRel (FinBERT)	72.89%	92.66%	81.26%
CasRel (FinBERT + PE)	78.57%	91.67%	84.62%

From the results, it can be seen that the introduction of FinBERT and position embeddings improved the performance significantly. It should be noted that although the F1-score reached 84.62%, it still means that some of the results have bias and errors from the actual situation. So, in practice, we added the confidence of the results as an attribute as well when we created the entities and relations in the knowledge graph to provide a reference for the user.

**Figure 4:**
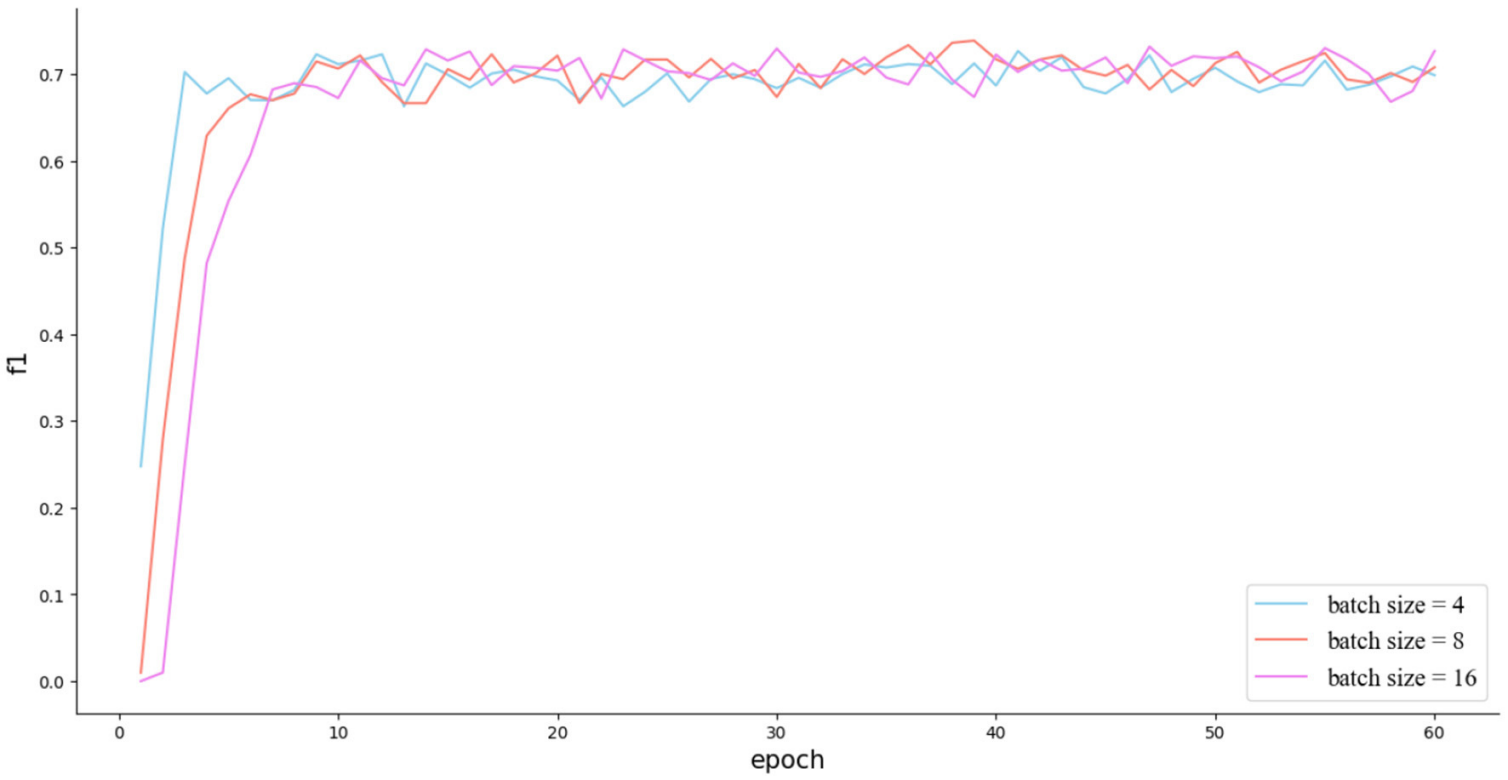
The impact of different batch sizes on the experimental results. In addition, it can be seen that the model has achieved the status of well-trained around 10 epochs.

Finally, we conducted experiments to investigate the effects of the batch size and learning rate. The batch size was set to 4, 8, and 16. The learning rate was set to 0.00001, 0.00005, and 0.0001. The experimental results, as shown in Figure 4, indicate that there was no significant gap in the effect regardless of whether the batch size was set to 4, 8, or 16. As for the learning rate, a larger learning rate was able to increase the convergence speed at the beginning, but a smaller learning rate made it easier to find the optima.

## 6. KGC: data fusion

After collecting multi-source heterogeneous data, data integration is required to combine and utilize all types of data. To generate well-integrated data, many data integration tools are commonly used, including Karma, D2R, and so on. These tools provide designable models for users to transform and normalize data in different structures. For the fusion of knowledge from different data sources, it is necessary to judge whether the new knowledge and the existing knowledge conform to the same data specification without conflict. That is, **Q3**: How to solve the data conflicts from different data source? should be considered. After the entities, relations, and attributes are obtained through information extraction, the new information can also be integrated into the original knowledge base through knowledge fusion to prevent redundant or wrong information.

In our practice, the constructed enterprise KG takes structured ERI data as the main information source and unstructured LRI and EAI as the secondary information sources. In other words, the current knowledge fusion is conducted mainly to integrate the enterprise entities and personnel entities extracted from the text, as well as the cooperation between them, into the enterprise KG. So, to link the extracted entities to the entities in the knowledge graph, we conducted candidate entity generation and candidate entity sorting.

### 6.1. Candidate entity generation

The generation of candidate entities mainly depends on the enterprise abbreviation mapping table and the method based on the character editing distance. We established the enterprise abbreviation name mapping table. For the enterprises in the graph database, their abbreviations can be divided into Chinese stock market security names (for example, “Huaxia Xingfu” corresponds to Huaxia Xingfu Jiye Co., Ltd.), English enterprise names (for example, “BOC” corresponds to Bank of China), field terms (for example, “AMC” corresponds to the Asset Management Company), and other abbreviations corresponding to the full name of the enterprise. Thus, the entity name abbreviations are restored before matching them to the ontology database. The method based on character editing distance is conducted to traverse the full name of the entity in the ontology database, compare it with the extracted entity for the character editing distance, and add the entities whose editing distances are less than the threshold value to the candidate entities. Character editing distance evaluation helps to select the entities that may be different from the full name of the enterprise due to having the wrong characters, character loss, and other reasons.

### 6.2. Candidate entity sorting

We adopted the popularity-based ranking method to rank the candidate entities. The basic assumption is that the more popular the candidate entity is, the more likely it is to be the target entity. Specifically, we queried through Wikipedia and search engines to obtain all entities *e*_*j*_ and calculate the ratio of the number of candidate entities *e*_*i*_ in *e*_*j*_, denoted as formula 8. Then, we selected the candidate entity with the highest score to link to.


(8) $$\begin{array}{l} Popularity\left(e_{i}\right)=\frac{count\left(e_{i}\right)}{\sum_{e_{j}\in E_{m}}count\left(e_{j}\right)} \end{array}$$

The advantage of the popularity-based ranking method is that it is simple to implement, while the disadvantage is that it ignores the contextual information. However, the entities we sorted were all enterprise names for which the context provides limited help to the field of the enterprise to be linked and has little impact on the results of the actual experiment. Therefore, we adopted the popularity-based method.

## 7. KGC: data storage

An important challenge arising from multiple sources of data is storage. Most enterprise KGs tend to use a graph databases for data storage, which is more flexible and suitable for graph data, but the limitation is that it does not take full advantage of other types of databases, for example, the execution time and memory usage of a relational database are both less than those of graph databases (Sholichah et al., 2020). Due to the diversity of enterprise data types and the sheer volume of data, based on our experience, it is a good choice to use a graph database to store triple data as the core and to use relational databases and the Elasticsearch database to store existing structured data and documents, thus enabling the need for interactions with multiple scenarios. Thus, in this section, to answer textbfQ4: How to select proper database to store data?, we first introduce how we use multiple databases in our enterprise KG and then provide a detailed evaluation of four typical graph databases for further comparison and reference.

### 7.1. Relational database

It is very straightforward to construct a knowledge graph from structured data, which are often stored in a relational database, i.e., MySQL. With its advantages in storage efficiency and query efficiency, relational databases can effectively solve the problem of one-step queries, e.g., querying all basic information from one enterprise. More importantly, since we need to frequently retrieve ERI data from external relational databases to update the knowledge graph, retaining a relational database would be more conducive to conducting an information comparison and update efficiency.

In our practice, first, we needed to confirm the required fields in the database table. Then, we queried the information from the API by entity name to obtain the basic information about the enterprise, and the “isComplete” field of the queried entity was labeled as 1. Since some enterprises related to the queried enterprise may also be collected, which we call derived enterprises, we labeled the “isComplete” fields of these derived enterprises/entities as 0. This means that if the “isComplete” field is 0, the enterprise entity is derived from other entities, and there is no independent data acquisition process for it. So, in that case, the basic information about this enterprise should be acquired by accessing the API again, and the relevant enterprise registration information for it will be added.

Specifically speaking, for ERI data, thirty-five MySQL database tables were constructed for data transfer according to the 35 fields of the data queried. For data involving inter-enterprise, person-enterprise, and inter-personal relationships, this can improve the usability of multi-hop graph queries by transferring them to the graph database.

From a general perspective, when using relational databases to store graph data, they are usually stored in the form of a triple table (representing graph data as RDF triples, with each triple as a row record in the table), a horizontal table (storing all predicates and objects corresponding to a subject in each row), and an attribute table (storing different types of entities in different tables). At the same time, join operations are used in relational databases to complete multi-hop queries.

### 7.2. Graph database

Although relational databases perform well for one-step queries, their response times for multi-step queries become significantly longer as the step increases due to the joint operations. In such cases, a graph database is more efficient as it can provide a quick multi-step query and search, such as querying the potential path between two enterprises within five steps. Different from relational databases, graph databases store data in a graph structure, i.e., nodes and edges. It is a very direct and natural way to model the data in the knowledge graph. In addition to efficient query of multi-step data, most graph databases are embedded with native algorithms and have their own graph query language, which make them easy to use.

In our practice, for EAI data, after natural language processing, the unstructured texts are transformed into triples of enterprise–relations–enterprise and person–relation–enterprise. These triples, rich in relational information, are stored in a graph database.

### 7.3. Elasticsearch

ElasticSearch is a Lucene-based search server. It provides distributed multi-user and full-text search services. Thus, in our practice, ElasticSearch is exploited to store the semi-structured long text, i.e., LRI data, and further search information from the stored data.

There are two main reasons to use Elasticsearch as our third database. The first reason is that the retrieval of the verdict documents should support text matching on the condition of word segmentation, which may help us find the required enterprise entities quickly (Xin et al., 2022).

The second reason is that when updating the verdict documents, we have to pull and compare all the data one-by-one. Since Elasticsearch is a more efficient way to retrieve a single piece of data, its performance is better in the process of comparing by unique identifiers. Thus, it becomes the first stop for the LRI data.

In all, a flexible storage architecture is designed to effectively solve the complex problems of multi-source data storage and updating. Different data sources choose different primary storage methods according to the characteristics of the data and realize their construction pipelines, reducing the coupling between different data sources.

### 7.4. Database evaluation

#### 7.4.1. Typical graph databases

In the process of constructing an enterprise knowledge graph from scratch, we tried many different graph databases. To provide a reference for subsequent research and practice, we evaluated four graph databases: Neo4j, ArangoDB, TigerGraph, and HughGraph. Next, we give a short introduction about them.

(1) Neo4j, an open-source NoSQL graph database developed on Java, ranks first on the Graph DBMS Ranking website^3^ with a community edition and a commercial edition. It is the most popular graph database with the largest number of users. (2) ArangoDB, a native multi-model database, is widely used and has more than 10,000 stars on Github. (3) TigerGraph is a real-time local parallel graph database, which can realize more than 10 steps of large graph traversal and performs well with multi-step queries. (4) HughGraph is an open-source graph database of Baidu company.

#### 7.4.2. Evaluation results

First, we conducted an experiment to evaluate four graph databases and record the performance indicators for each graph database. It was evaluated on a flight dataset^4^ provided by ArangoDB with 3,375 nodes and 286,463 edges. They were all deployed on an eight-core server [Intel(R) Xeon(R) CPU E5-2680 v4 @ 2.40 GHz] with 32 GB of RAM. The main results are shown in the first block of Table 5. TigerGraph and HugeGraph took the shortest amounts of time to batch import data, while HugeGraph lagged behind the other databases when importing single data. Besides the performance, we list some metrics, such as supported programming languages, whether open-source or not.

**Table 5:** The evaluation of four typical graph databases.

**Dimension**	**Neo4J**	**ArangoDB**	**TigerGraph**	**HugeGraph**
Time taken to batch import data	2 s	10 s	0–10 s	0–10 s
Time taken to import single data	0–10 ms	0–10 ms	0–10 ms	0–1 s
Traversing the KG	0–10 ms	0–10 ms	0–10 ms	1,173 ms
Querying the shortest path	0–10 ms	0–500 ms	1–5 s	0–10 ms
Database query language	Cypher	AQL	GSQL	Gremlin
Supports Python language	✓	✓	✓	✓
Supports Java language	✓	✓	✓	✓
Supports JavaScript language	✓	✓	✗	✗
Open-source	Yes	Yes	No	Yes
Native algorithms	4	5	3	2
Distributed deployment	5	5	4	4
Data migration	4	4	4	3

The first block reports some objective metrics, and the second block reports three subjective metrics. Its values range from 1 to 5.

In addition to some objective metrics, we also conducted subjective evaluations. Specifically, we used the Delphi method to send questionnaires to the five technicians who participated in the construction of the knowledge graph. The purpose was to evaluate each database based on the subjective user experience. The respondents were asked to give a score of 1–5 based on their user experience, where 1 represents a poor experience, 5 represents a very satisfactory experience, and 3 represents not good or bad. The questionnaire consisted of three items: the availability of native algorithms, the ease of use for distributed deployment, and the convenience of data migration. The main results are shown in the second block of Table 5.

In summary, graph databases and relational databases have their pros and cons, so a mixture of multiple databases may be a good practice for practical applications, not only for ease of access but also to improve the retrieval efficiency. As for the selection of a graph database, the four graph databases have their advantages and disadvantages. Neo4j,^5^ as the top graph database for many years, has a stable performance, rich documentation, and an active community; ArangoDB ^6^ is currently one of the best open-source graph databases in terms of performance, but its commercial application in the Chinese market is seldom; and TigerGraph,^7^ as a rising star in the graph database, is roughly equal to Neo4j in terms of performance and ease of use, and has special optimization for multi-hop.

As for the selection of a graph database, the four graph databases have their advantages and disadvantages. Neo4j, as the top graph database for many years, has a stable performance, a rich documentation, and an active community; ArangoDB supports many programming languages and provides a good user experience for technicians; and TigerGraph, as a rising star in the graph database, has a comparable performance and SQL-like query language. Although we chose TigerGraph as our graph database, all the listed databases can satisfy the need to construct a medium-size knowledge graph.

## 8. KGC: data update

The knowledge graph constructed originated from three data sources, ERI, LRI, and EAI, which all dynamically change over time, so after the KG construction is finished, it will become out-of-date gradually, and the life cycle of the KG is very limited. To ensure the real-time nature of the KG, **Q5**: When and how to update the data in the KG is raised, so a suitable update strategy needs to be designed.

The update strategy we adopted is a combination of passive updates and active updates. Redis was used as the storage container for the enterprise entities that needed to be updated, and scheduled tasks were set up to read the enterprise entities in Redis and update the knowledge graph data regularly. The combination strategy avoids repeated updates and improves the efficiency, tries to solve the problem of untimely updates of the associated data at the depth level of the graph, and ensures that the timeliness of the data is transparent to users.

### 8.1. Active update strategy

From the perspective of system user roles, active updates can be initiated by ordinary users or administrators. For ordinary users, when querying an enterprise, the user can obtain the last update time of the enterprise data through the returned field “lastUpdateTime”. If the last update time of the data does not meet expectations, the user can initiate an update application. Moreover, if the queried enterprise does not exist in the KG, users can also initiate an application to add information about the enterprise. To avoid malicious or high-frequency applications, this kind of application cannot be directly added to the Redis update queue but needs to be reviewed by the administrator. Such an interaction makes it easier to understand user needs.

Administrators are responsible for handling all applications initiated by users and deciding whether to agree or reject them. Once the applications have been approved, the queried enterprises will be added to the Redis update queue. In addition, the administrator can also update the existing enterprise information and add new enterprises to Redis in batches, which is designed to make up for the shortcomings of passive updates, such as the inability to check in-depth data and the inability to add new data.

### 8.2. Passive update strategy

Passive updates refer to periodic automatic checks. A data timeliness checking module is added to ensure the validation of data. Take the ERI data update as an example. After obtaining the data from the API, we compared it with the enterprise information stored in MySQL. If there was a difference, we checked whether the enterprise already existed in Redis; if not, we pressed it directly into Redis. Passive updates do not require human processing, and the timeliness of the data is automatically checked and updated during the use of the system, which not only saves labor costs but also ensures the timeliness of the data.

## 9. Case study: corporate due diligence application on the enterprise knowledge graph

Following the above steps, we constructed a medium-sized enterprise knowledge graph with 1,430,000 nodes and 3,170,000 edges. Then, we implemented a Corporate Due Diligence System, which aims to provide various data query functions from the perspective of the enterprise to assist business personnel in decision-making, collaborative analysis, and collaborative office. The specific business applications are summarized in Sections 9.2–9.5.

### 9.1. Background

Corporate due diligence is an important risk management tool in the financial domain, which includes a series of investigations conducted by the acquirer on the assets and liabilities, operating and financial situation, legal relations, and opportunities and potential risks faced by the target company during the acquisition process. It plays an important role in early risk control for investment, cooperation, and other decision-making processes.

**Figure 5:**
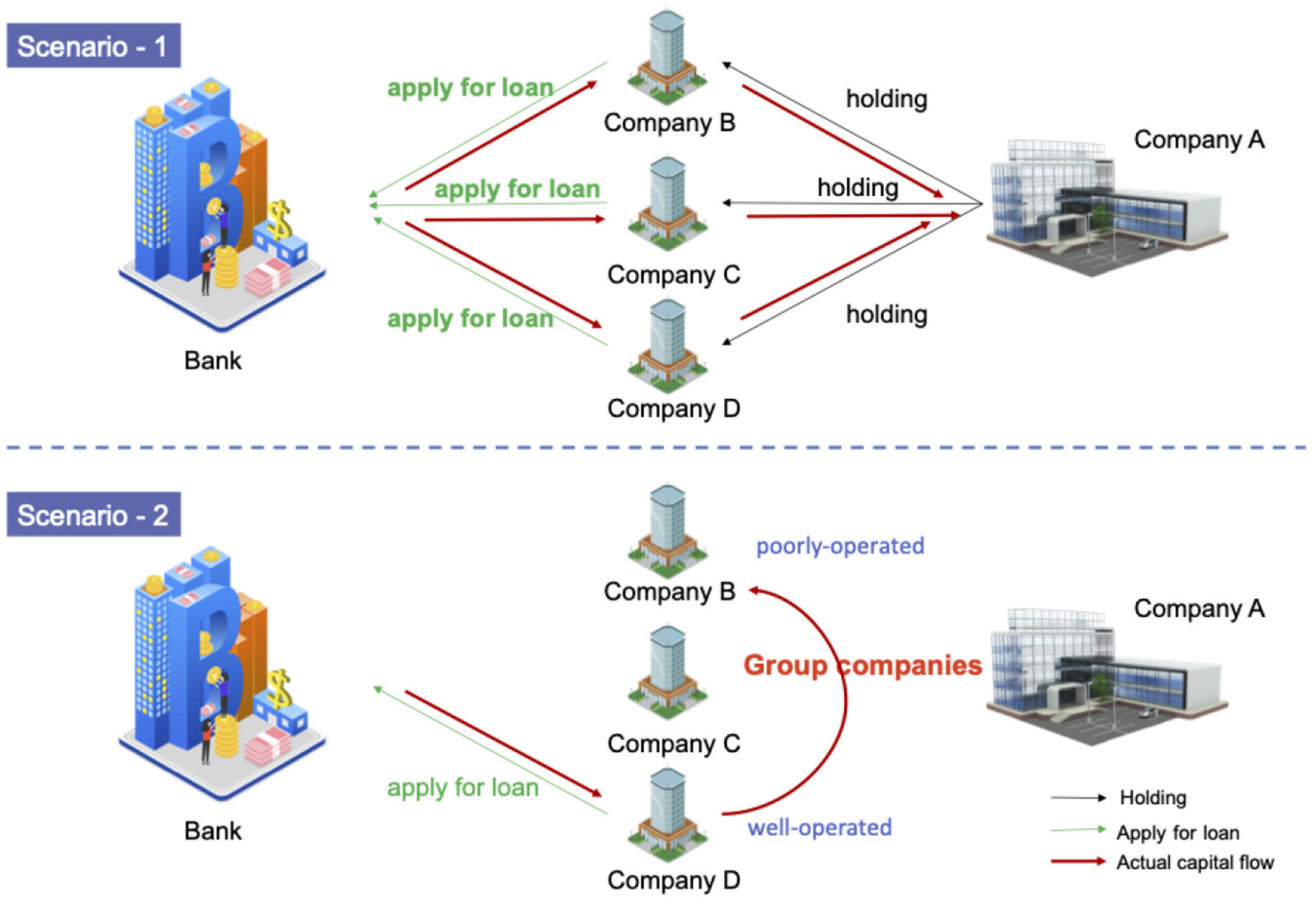
The scenarios of risk spread between enterprises, which have complex relationships with each other. In scenario 1, A, B, C, and D are the group companies, and A holds the other three companies. All the funds obtained by B, C, and D eventually flow to company A. In scenario 2, A, B, C, and D are still group companies, but B is a poorly operated company, and D is a well-operated company. Company D transfers the funds to company B, because it is difficult for B to obtain a loan in its own name. The red line shows the actual capital flow.

Figure 5 illustrates two typical scenarios that require thorough corporate due diligence. In scenario 1, B, C, and D apply for loans from the bank in their own names, but unfortunately the real user of the loan is company A. In scenario 2, the well-operated company D with good credit applies for a loan and uses the group transfer to help poorly operated company B. The real user of the loan is company B. These actions will bring a lot of risks to the bank. If the bank does not figure out the relationship between the companies and lends the money blindly, the consequences are unimaginable.

A similar case occurred in real life. Minsk Aircraft Industry Co., Ltd. (Minsk) was established in 1998, of which Delong International Strategic Investment Co., Ltd. (Delong) invested CNY 250 million, accounting for 89%. After Minsk obtained the loan from the bank successfully, the money was handed over to its parent company. When the Delong crisis broke out in 2004, Minsk was unable to pay off its due debts, and several banks applied to the court for bankruptcy and debt repayment. According to the court's investigation, Minsk's total assets were CNY 670 million, and the amount of insolvency was CNY 190 million.

Thus, it is critical for the bank to have better risk management, and corporate due diligence is a necessity. The risk identification process requires the collection of comprehensive data from the company for analysis and evaluation. Faced with a large amount of data from multiple sources, business personnel need to spend a lot of time and energy to collect high-quality information, and traditional structured data are difficult to mine for deep association information. Also, corporate due diligence involves many fields, such as law, finance, business, etc., and the use of multi-source information in different formats is a necessity. The core idea is determining how to effectively organize and make full use of these data. Fortunately, the emergence of the concept of a knowledge graph provides an opportunity to solve this situation.

### 9.2. Single-point graph query

The graph query of the single-point graph is the basic function. The query logic is to start from an enterprise entity, and search and show all of the entities and relations within *n* steps. We only consider nodes connected by one-way edges starting from the source enterprise, and *n* is usually assigned a value of three due to the trade-off between efficiency and query results.

**Figure 6:**
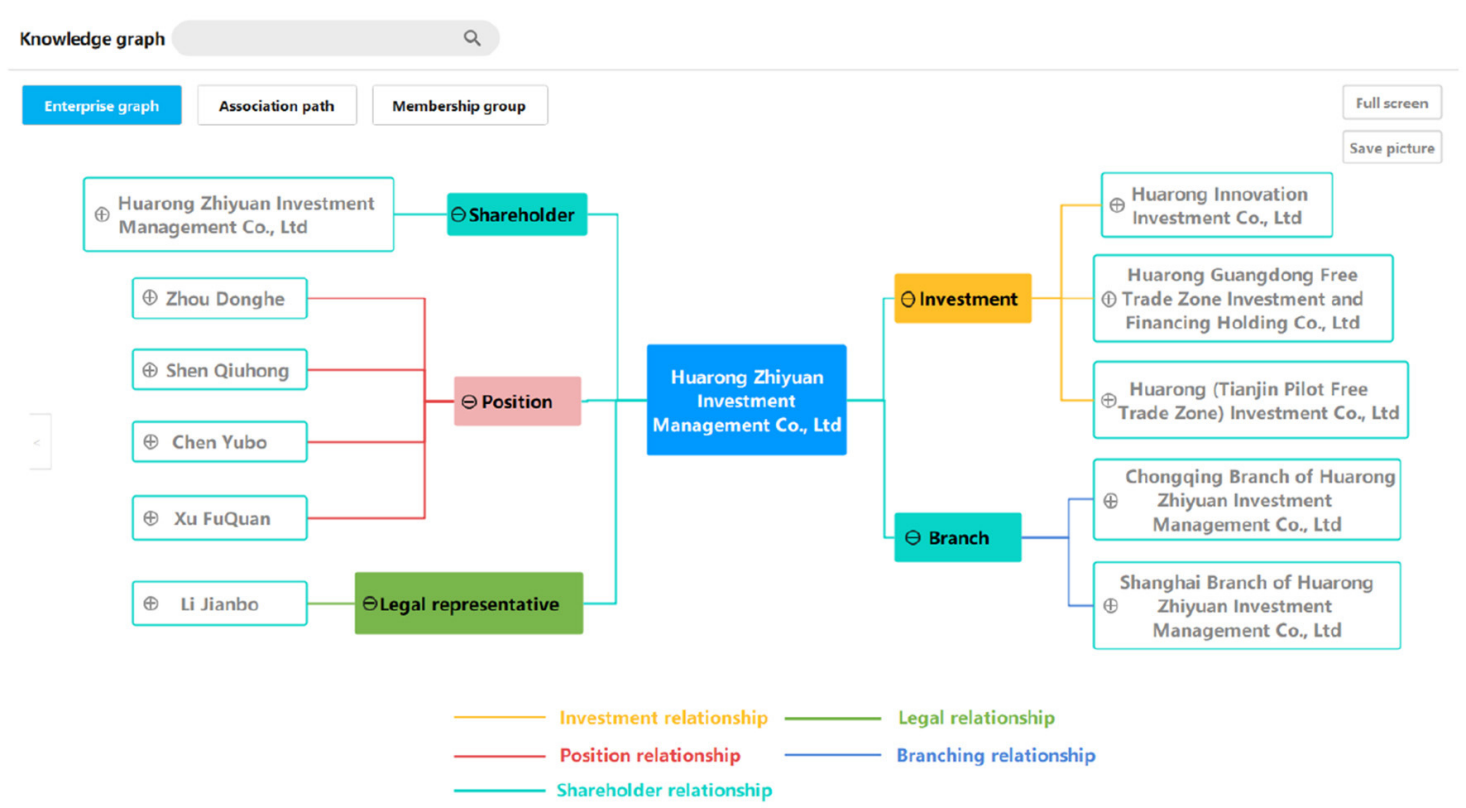
A query result example of a single-point graph.

As for the virtualization of the query results, as shown in Figure 6, a single-point graph is a one-way map centered on the source enterprise. Moreover, the display can be manually selected during the interaction, and the user can simplify the tree-based map to focus on the required information.

### 9.3. Association path query

**Figure 7:**
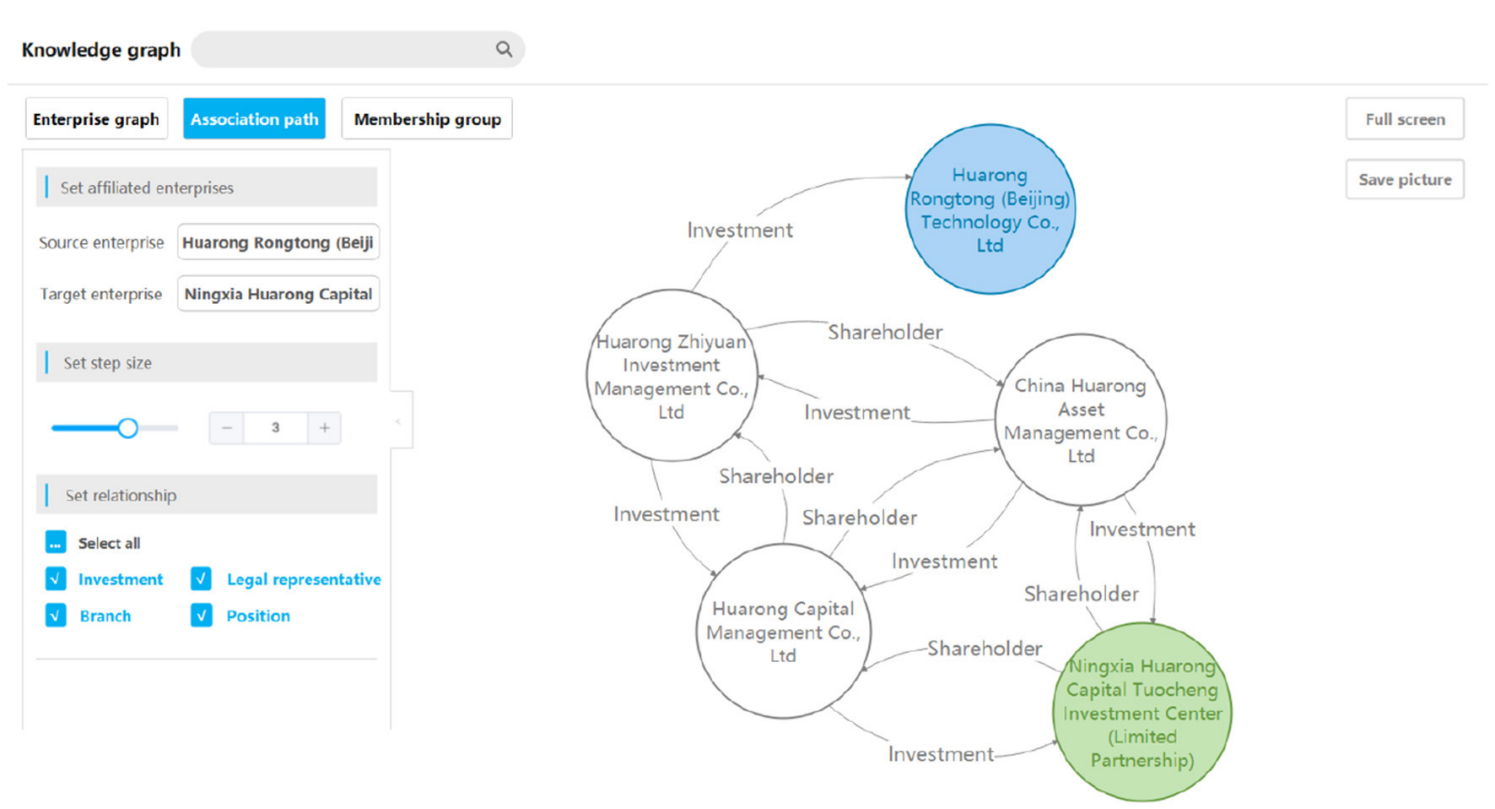
A query result example of an association path.

The query of the association path refers to finding all association paths between two enterprises, as shown in Figure 7, which can dig deep into the potential relationship information hidden in the data.

This function requires users to enter the names of the two enterprises and the largest step *n*_*max*_. The results return all paths from enterprise A to enterprise B within *n*_*max*_ steps.

Another query is the revolving investment query, which is used to find out whether an investment loop exists around the queried enterprise. We used the Cypher statement to find all the two-way investment edges of the queried enterprise within *n* steps. After obtaining the returned graph data, the Depth First Search (DFS) algorithm was used to find all the loops by regarding the queried enterprise as the starting node.

### 9.4. Enterprise group query

**Figure 8:**
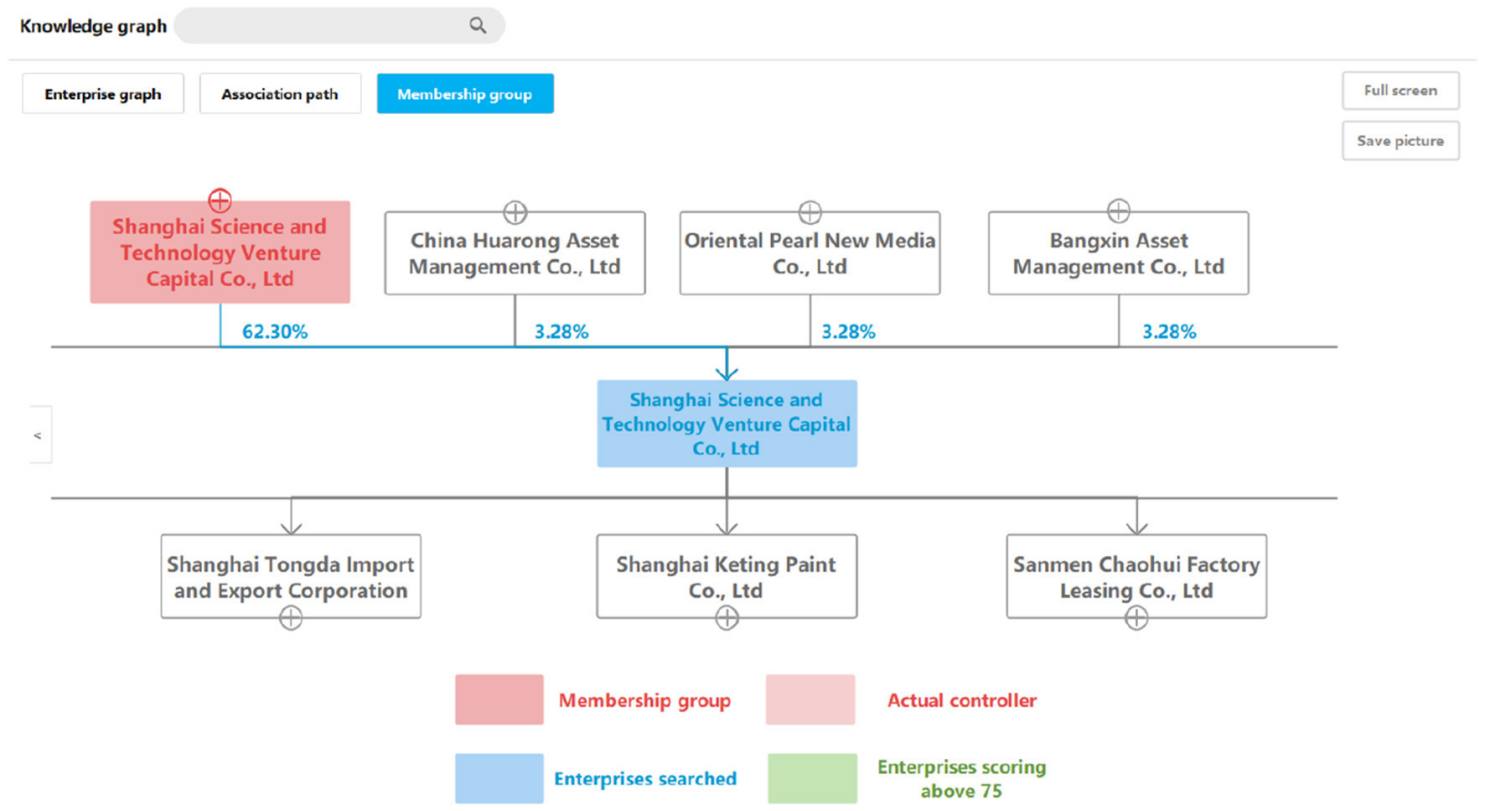
A query result example of an enterprise group graph.

The enterprise group query aims to find the enterprise with the highest shareholding ratio of the queried enterprise within 10 steps. First, we used the Cypher statement to find all shareholders from the queried enterprise within 10 steps. Then, we calculated the true shareholding ratio based on data from the attribute value to find the one with the highest actual shareholding ratio. The true shareholding ratio is the sum of direct holdings and indirect holdings. The calculation here uses the method of the cross-shareholding calculation to improve the efficiency. Figure 8 illustrates a true example of this kind of query.

### 9.5. Guarantee circle query

**Figure 9:**
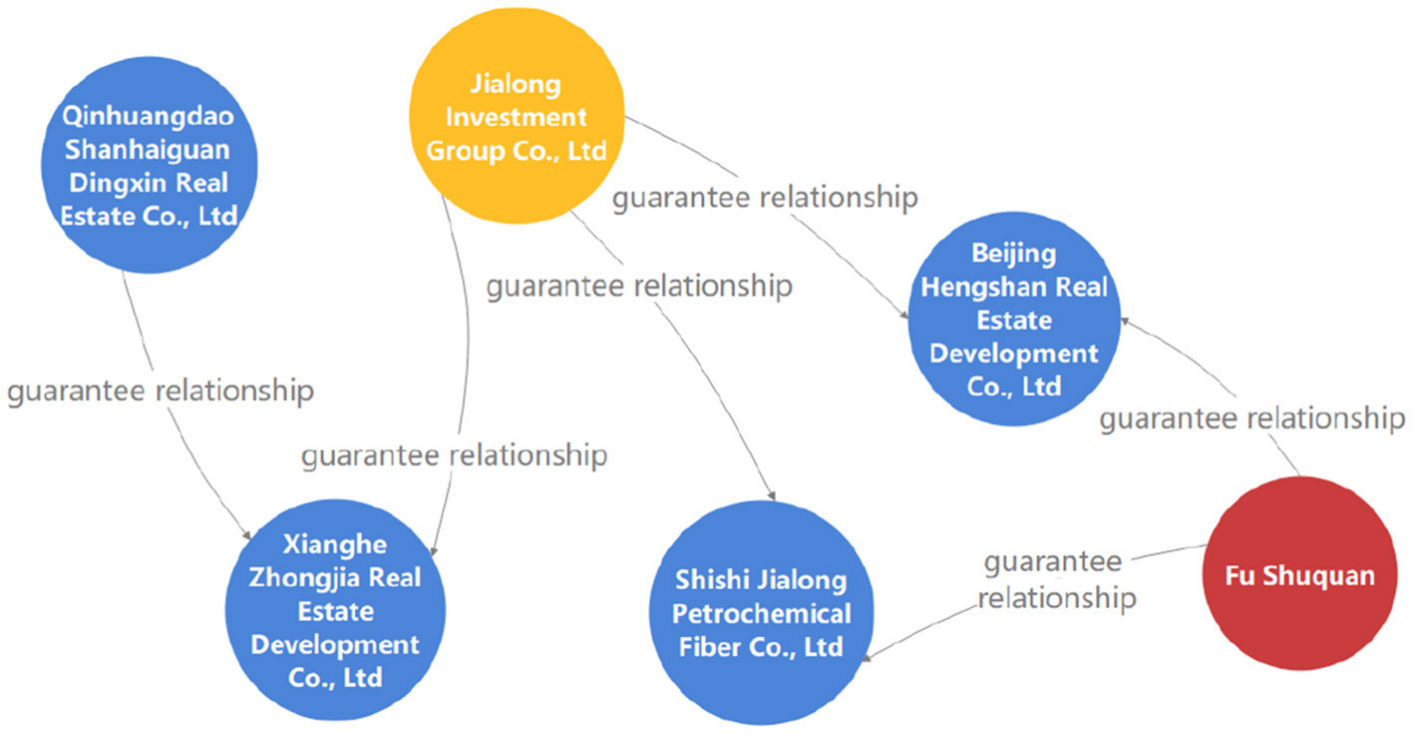
A query result example of revolving investment.

The guarantee circle refers to a special interest entity with a guaranteed relationship as a chain formed by multiple enterprises connected by mutual guarantee or serial guarantee. As the scale of commercial banks' assets continues to grow, guaranteed loans also increase, and mutual guarantees or chain guarantees between customers form guarantee circle loans. As shown in Figure 9, the formation of the guarantee circle causes the originally unconnected companies to become closely related. The risk of one enterprise in the circle will spread along the guarantee chain, triggering the loan risk of other enterprises in the circle.

## 10. Discussion and conclusion

In general, there is an urgent need in the financial field to clarify the complex relationships between enterprises. The emergence of the concept of knowledge graph provides an opportunity to solve this situation. The knowledge graph, as a common technique used in the smart finance domain, can integrate multi-source data in the form of entity relations. Relying on the stored data of the graph structure, graph theory algorithms can be used to mine the potential information from the data and assist business personnel in decision-making.

What's more, benefiting from graph structure data format and the multi-source data, our Corporate Due Diligence System can efficiently utilize graph theory algorithms to mine the hidden information (Bernardete et al., 2019) between data and assist the due diligence personnel to make decisions.

Thus, from a practical perspective, this paper provides a solution for the construction of enterprise knowledge graphs. We constructed an enterprise knowledge graph that integrates multi-source data, including enterprise register information (ERI), litigation-related information (LRI), and enterprise announcement information (LRI). It aims to solve the difficulties of data acquisition, mining, and analysis and improves the time-consuming and laborious status of business personnel in the traditional business model. Furthermore, from a management perspective, benefiting from the graph's structural data format and the multi-source data, our corporate due diligence system can efficiently utilize graph theory algorithms to mine the hidden information (Bernardete et al., 2019) between data and assist the due diligence personnel to make decisions. It has significant implications for risk control. In terms of broader management significance, when facing tasks in finance or other fields, it is also possible to consider a knowledge graph-based approach. By following the framework proposed in Section 3 and following the design and development process of Sections 4-8, management efficiency can be improved and new tasks can be applied.

### 10.1. Limitations

One limitation of our enterprise knowledge graph and its application is that the update strategy does not consider priority issues. The current update queue, which uses Redis queue management, operates on a first-come, first-served basis, i.e., the enterprise to be updated that has been pressed into Redis will trigger the update first. If the enterprise to be updated is already in the queue, it will not be pressed repeatedly. This may cause some enterprises that need urgent updates to not be updated quickly. In follow-up work, priority should be given to designing a more comprehensive update queue data structure, such as adding the waiting time, number of applications, and other indicators.

## Data availability statement

The raw data supporting the conclusions of this article will be made available by the authors, without undue reservation.

## Ethics statement

Ethical approval was not required for the study involving human participants in accordance with the local legislation and institutional requirements. Written informed consent to participate in this study was not required from the participants in accordance with the national legislation and the institutional requirements.

## Author contributions

CY: Methodology, Writing—original draft, Writing—review and editing, Conceptualization, Formal analysis, Software. XF: Investigation, Writing—review and editing, Formal analysis, Validation. XH: Investigation, Validation, Writing—review and editing, Conceptualization, Software. CG: Writing—review and editing, Supervision, Validation, Funding acquisition. JW: Supervision, Writing—review and editing.

## Footnotes

^1^ https://s.ccxe.com.cn/entities/companies

^2^ http://www.aicubes.cn/api/public/home.html

^3^ https://db-engines.com/en/ranking/graph+dbms

^4^ https://www.arangodb.com/graphcourse_demodata_arangodb-1/

^5^ https://db-engines.com/en/system/Neo4j

^6^ https://db-engines.com/en/system/ArangoDB

^7^ https://db-engines.com/en/system/TigerGraph
